# Effects of High‐Dose Multichannel Radiofrequency Treatment on Visceral and Subcutaneous Abdominal Fat: A Pre–Post Interventional Study

**DOI:** 10.1111/jocd.71051

**Published:** 2026-07-09

**Authors:** Jesus Rodríguez‐Lastra, Sidra Kouser, José Rioja, Rosa María Ordóñez‐Simón, Antonia Blanch, Santiago Mourelo, José Antonio Romero, Javier Espíldora‐Hernández, Miguel Ángel Sánchez‐Chaparro

**Affiliations:** ^1^ Department of Physiotherapy. School of Health Sciences Universidad Alfonso X el Sabio Villanueva de la Cañada Madrid Spain; ^2^ INNEOTERAPIA Barcelona Spain; ^3^ Lipid and Atherosclerosis Laboratory (CIMES), Department of Medicine and Dermatology University of Málaga Málaga Spain; ^4^ IBIMA Plataforma BIONAND Instituto de Investigación Biomédica de Málaga Málaga Spain; ^5^ CETIR Medical Center S.L. Barcelona Spain; ^6^ Lipid and Hypertensión‐Vascular Risk Unit, Internal Medicine Service Virgen de la Victoria University Hospital Málaga Spain

**Keywords:** adipokines, insulin resistance, intra‐abdominal fat, radiofrequency therapy, subcutaneous fat

## Abstract

**Objectives:**

Energy‐based devices (EBDs) have been used as a radiofrequency treatment for abdominal obesity, although their effects on visceral fat are not well known. This study aimed to evaluate the impact of applying an EBD treatment on visceral and subcutaneous adipose tissue, anthropometric values, lipid profile, adipokines, and the insulin resistance index.

**Methods:**

**We conducted a prospective** pre–post interventional study in a single outpatient clinical body‐contouring facility in Barcelona, Spain. Twenty adults (10 men, 10 women; mean age 49 ± 11 years) with BMI 25–< 30 kg/m^2^, abdominal obesity, and visceral fat score > 9 were treated with EBD‐radiofrequency using the Endoxslim device: 10 sessions of 60 min plus 20 min with capacitive and resistive electrodes at temperature > 45°C. We analyzed anthropometric changes, and visceral and subcutaneous fat were quantified by magnetic resonance imaging. Serum samples were obtained at the beginning and end of treatment for the quantification of the biochemical variables.

**Results:**

At the end of the treatment period, patients significantly reduced waist circumference, systolic blood pressure levels, and visceral and subcutaneous fat mass (*p* < 0.05). In addition, leptin levels, basal insulinemia, and HOMA‐IR index were also reduced (*p* < 0.05). No adverse effects were reported.

**Conclusions:**

EBD is a safe and effective method to reduce subcutaneous and visceral fat in abdominal obesity, with consequent improvement in the metabolic profile. ClinicalTrials.gov Identifier: NCT06377358.

## 1 Introduction

The prevalence of obesity has increased globally in recent decades, along with its association with insulin resistance, cardiometabolic problems, and cancer. In addition to these metabolic consequences, many individuals experience dissatisfaction with obesity and its aesthetic implications [1, 2, 3]. In this context, non‐invasive body‐contouring modalities, particularly those targeting abdominal obesity, have gained popularity for addressing aesthetic concerns while avoiding surgical risks.

Complementary therapies have gained interest as adjuvant strategies to improve body composition and modulate risk factors associated with chronic diseases. These approaches have been applied in trials with natural compounds [4, 5] that seek to modulate inflammatory and metabolic processes, suggesting a potential complementary role to conventional interventions.

Energy‐based devices (EBDs) that deliver high‐frequency electromagnetic energy have also been used for abdominal fat reduction. They are relatively low‐cost and generally well‐tolerated. When applied to tissue, oscillating electromagnetic fields induce motion of electrically charged particles, generating heat (42°C–45°C). The depth and magnitude of heating depend on the tissue's bioimpedance. This thermal energy upregulates adipose triglyceride lipase (ATGL) and hormone‐sensitive lipase (HSL), promoting triglyceride hydrolysis into glycerol and free fatty acids (FFAs) [6]. These mechanisms contribute to reductions in subcutaneous fat volume and improvements in skin elasticity, with effects sustained for up to 6 months [7]. Additionally, thermal energy activates sympathetic fat innervation, triggering catecholamine release and further stimulating lipolysis and lipid efflux [8].

More intense or prolonged thermal exposures can induce adipocyte apoptosis and, at higher thresholds, necrosis. In vitro and ex vivo studies show loss of membrane integrity at 43°C–45°C within minutes and delayed adipocyte death after 15 min at these temperatures [9, 10].

These findings may explain the reductions in visceral adipose tissue (VAT) and subcutaneous adipose tissue (SAT) observed in treated subjects [7, 11].

Visceral adipose tissue is widely recognized as a principal driver of insulin resistance and an independent predictor of adverse cardiovascular outcomes. It contributes to metabolic dysfunction–associated steatotic liver disease (MASLD), atherogenic dyslipidemia, endothelial dysfunction, hypertension, and chronic low‐grade systemic fibro‐inflammation [12, 13, 14]. Emerging evidence indicates that certain radiofrequency techniques, alone or combined with ultrasound and accompanied by exercise and diet, may improve visceral adiposity, leptin levels, lipid profiles, and insulin resistance. Conversely, abdominal and truncal SAT appear to exert comparatively less influence on insulin resistance and metabolic syndrome than VAT [15].

We hypothesize that selective adipocyte loss induced by EBD treatment may confer metabolic benefits. This study aims to evaluate the effects of EBD on MRI‐quantified VAT and SAT, as well as on anthropometric measures, lipid profile, adipokines, and insulin resistance. These effects have not been previously demonstrated with other techniques (e.g., liposuction); if confirmed, this would justify larger‐scale trials.

## 2 Methods

### 2.1 Study Design

The study was designed as a pilot pre‐post interventional study with the primary objective of evaluating changes in visceral and subcutaneous abdominal fat after a course of high‐dose multichannel radiofrequency treatment. We chose this design for practical and ethical reasons: in the initial phase, we sought to estimate the magnitude of the effect and safety before investing in a randomized controlled trial (RCT). The observed average loss of approximately 1.3 kg of abdominal fat in the period evaluated is clinically relevant.

### 2.2 Patients and Treatments

Adults aged 20–80 years with BMI 25–< 30 kg/m^2^, abdominal waist circumference > 102 cm (men) or > 88 cm (women), and visceral fat score > 9 on a 0–20 scale [16] were eligible. Participants were recruited consecutively from among individuals seeking body‐contouring treatment at the clinic. Exclusion criteria included inability or unwillingness to provide written informed consent; contraindications to EBD (e.g., pregnancy, metallic implants/prostheses, active infection); history of malignancy or ongoing chemotherapy/radiotherapy; diabetes mellitus; other metabolic disease; or arterial hypertension.

The minimum sample size (15 patients) for paired data was calculated using Epidata Software (EpiData, Odense, Denmark) based on previous studies [Photochem Photobiol B Biol. 2015;149:21–26]. The sample comprised 20 participants (10 men, 10 women) enrolled consecutively. Prior to treatment, participants received standardized instruction on technique and safety precautions and were asked to report unexpected increases in perceived temperature.

Recruitment began on 3 February 2022; of 32 individuals screened, 12 were excluded (five for BMI ≥ 30 kg/m^2^, seven for diabetes). The final cohort (*n* = 20) initiated treatment on 28 March 2022 and completed it on 3 June 2022. All participants belonged to a single intervention group with no randomization or blinding, consistent with a pre–post design. The study was registered at the https://register.clinicaltrials.gov/ with the number NCT06377358.

Participants were treated in the supine position with the Endoxslim EBD device (Capenergy, Barcelona). Four emitting antennas (each 200 cm^2^) were secured, two on each side of the abdominal midline, using an adjustable belt. Delivery was automatically regulated according to abdominal energy absorption. Power was applied for 60 min (310 W per channel × 4 channels; 1240 W total), maintaining tissue temperature at 45°C–50°C under continuous monitoring. Participants were instructed to perceive only comfortable warmth. Afterward, 20 min of manual capacitive and resistive electrode application was performed on the abdomen. Simultaneously, 20 min of lymphatic drainage was delivered using two active plates placed on the plantar surfaces and passive plates positioned over the lumbodorsal region; based on magnetohydrodynamic principles [17, 18], this configuration facilitates mobilization of released substances.

The schedule comprised five sessions per week (Monday–Friday) for two consecutive weeks (10 sessions total). No dietary or exercise modifications were recommended, and participants were instructed to maintain their usual lifestyle.

### 2.3 Clinical, Anthropometric, and Laboratory Measures

Primary anthropometric outcomes were SAT and VAT (cm^3^ and g), measured by MRI. Secondary outcomes were waist‐to‐hip ratio and BMI (kg/m^2^). Measurements were taken at baseline and after treatment. Waist circumference was measured at the midpoint between the lower rib and iliac crest using a standard tape measure. BMI was calculated from measured weight and height.

**FIGURE 1:**
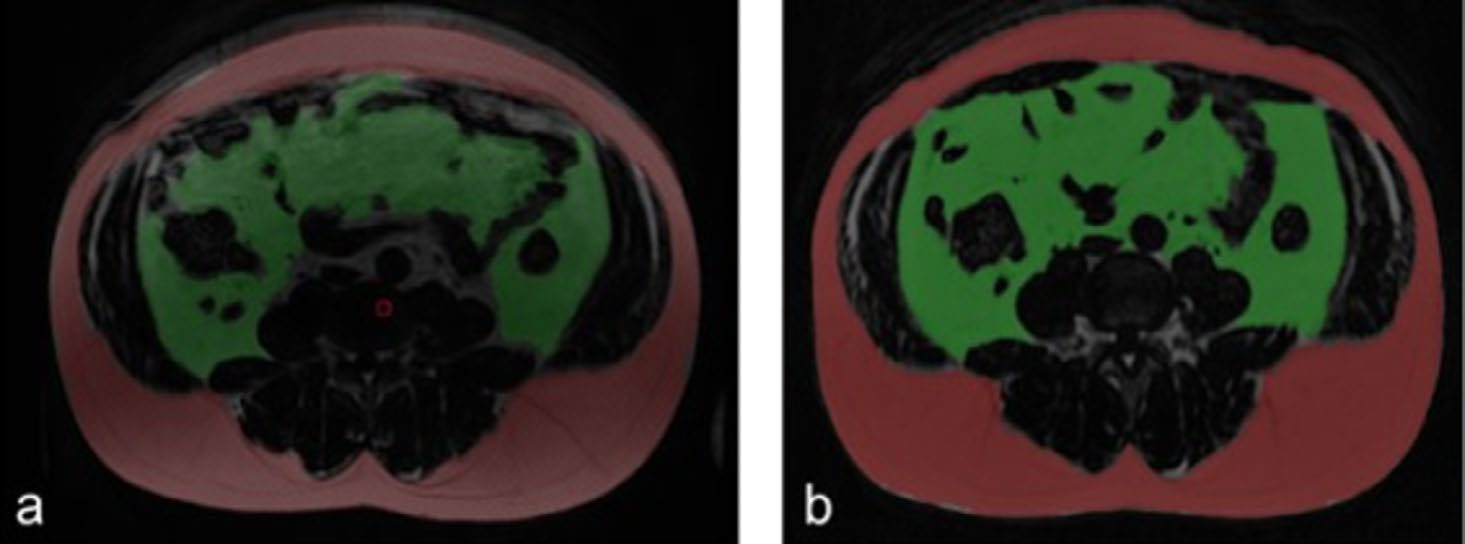
Magnetic resonance imaging showing the areas of visceral adipose tissue and subcutaneous adipose tissue.

MRI scans were performed on a General Electric 3 T Sigma Pioneer scanner (CETIR, Barcelona, Spain) using DIXON sequences with 15‐s breath‐holding intervals. The axial volume was acquired, and 160 slices (3 mm thickness) were reconstructed. Data were reconstructed using a standard single‐peak fat spectral model. Each sequence produced water, fat, in‐phase, and out‐of‐phase images (Figure 1). Volumetric segmentation of abdominal fat (from hepatic region to iliac crests) was performed using ITK‐SNAP software. VAT and SAT were manually analyzed without automatic interpolation. Measurements were performed by a biomedical engineer and reviewed by a radiologist.

After a 12‐h fast, blood samples were collected in serum and EDTA‐K2 tubes. Serum samples were kept for 30 min at room temperature before centrifugation at 3000 rpm for 15 min at 4°C. Aliquots were frozen at −70°C.

Triglycerides, total cholesterol, LDL‐C, and HDL‐C were measured by enzymatic methods using the Atellica CH 950 analyzer (Siemens, Germany). Non‐HDL cholesterol was also calculated.

Plasma IL‐6 and TNF‐α were measured by chemiluminescent ELISA (R&D, Germany). High‐sensitivity C‐reactive protein was measured by immunoturbidimetry (Spinreact, Spain) on a Mindray BS‐380 analyzer.

Adiponectin, resistin, and leptin were measured by ELISA (MEDIAGNOST, Germany). Serum glucose and insulin were measured for HOMA‐IR calculation.

### 2.4 Statistical Analysis

Statistical analysis was performed using SPSS Statistics (version 28.0, IBM, USA). Normality of the data distribution was assessed using the Kolmogorov–Smirnov (K‐S) test. Continuous variables were expressed as mean ± standard deviation for normally distributed data (*p* value for K‐S ≥ 0.05) or as median (percentile 25–percentile 75) for non‐normally distributed variables.

To evaluate the impact of the RF intervention, pre‐ and post‐treatment values were compared using the paired‐samples *t*‐test for variables following a normal distribution, while the Wilcoxon signed‐rank test was applied for variables that did not meet normality. Categorical variables were expressed as frequencies and percentages and compared using the Pearson chi‐squared test or Fisher's exact test, as appropriate. A *p*‐value of < 0.05 was considered statistically significant.

### 2.5 Ethical Aspects

The study was approved by the Ethics Commission in Animal and Human Experimentation of the Universitat Autònoma de Barcelona (25 February 2022; ref. 5324). All participants provided written informed consent.

## 3 Results

Twenty patients participated (10 men, 10 women), aged 49 ± 11 years.

**FIGURE 2:**
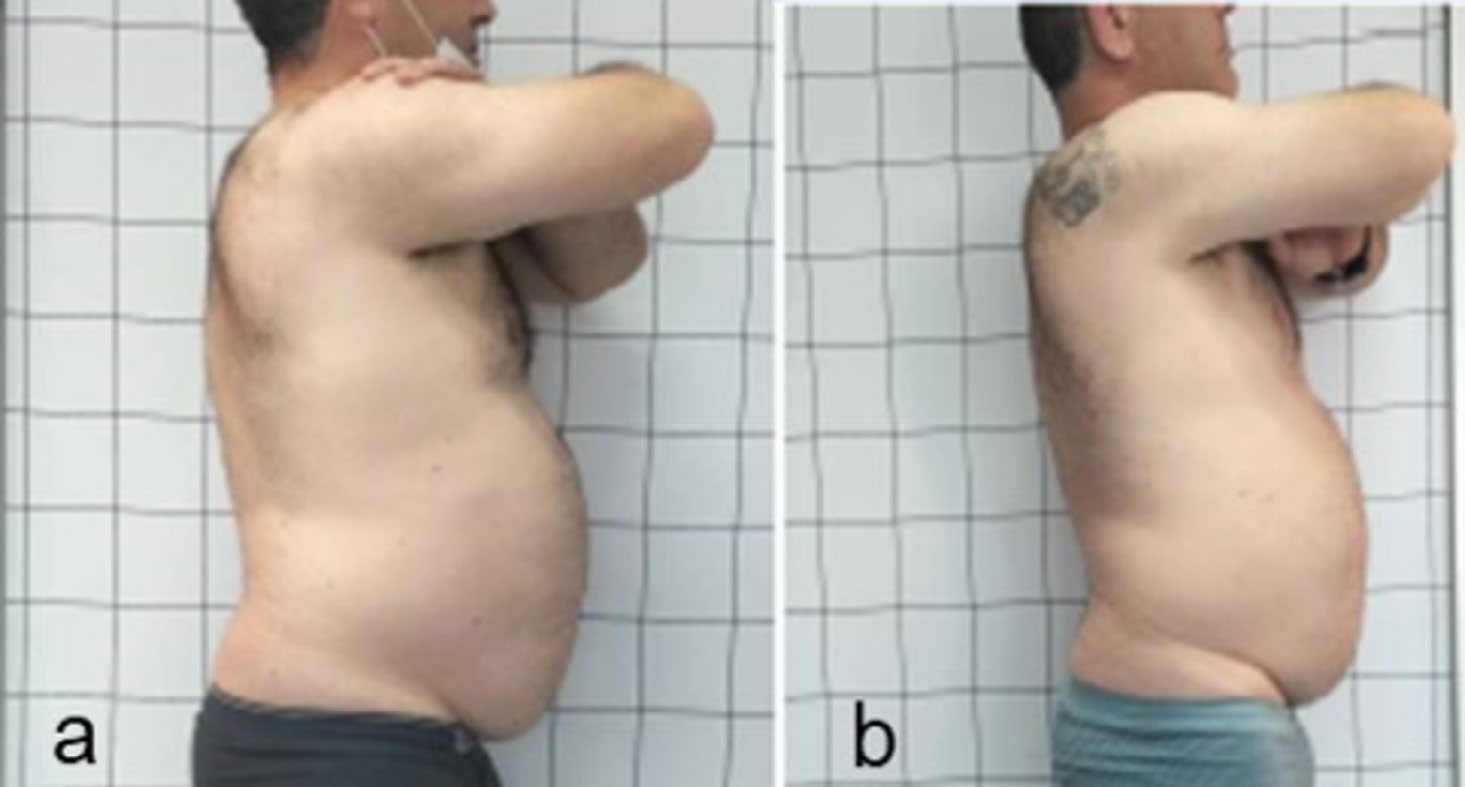
Patient showing abdominal obesity, before and after the procedure.

**FIGURE 3:**
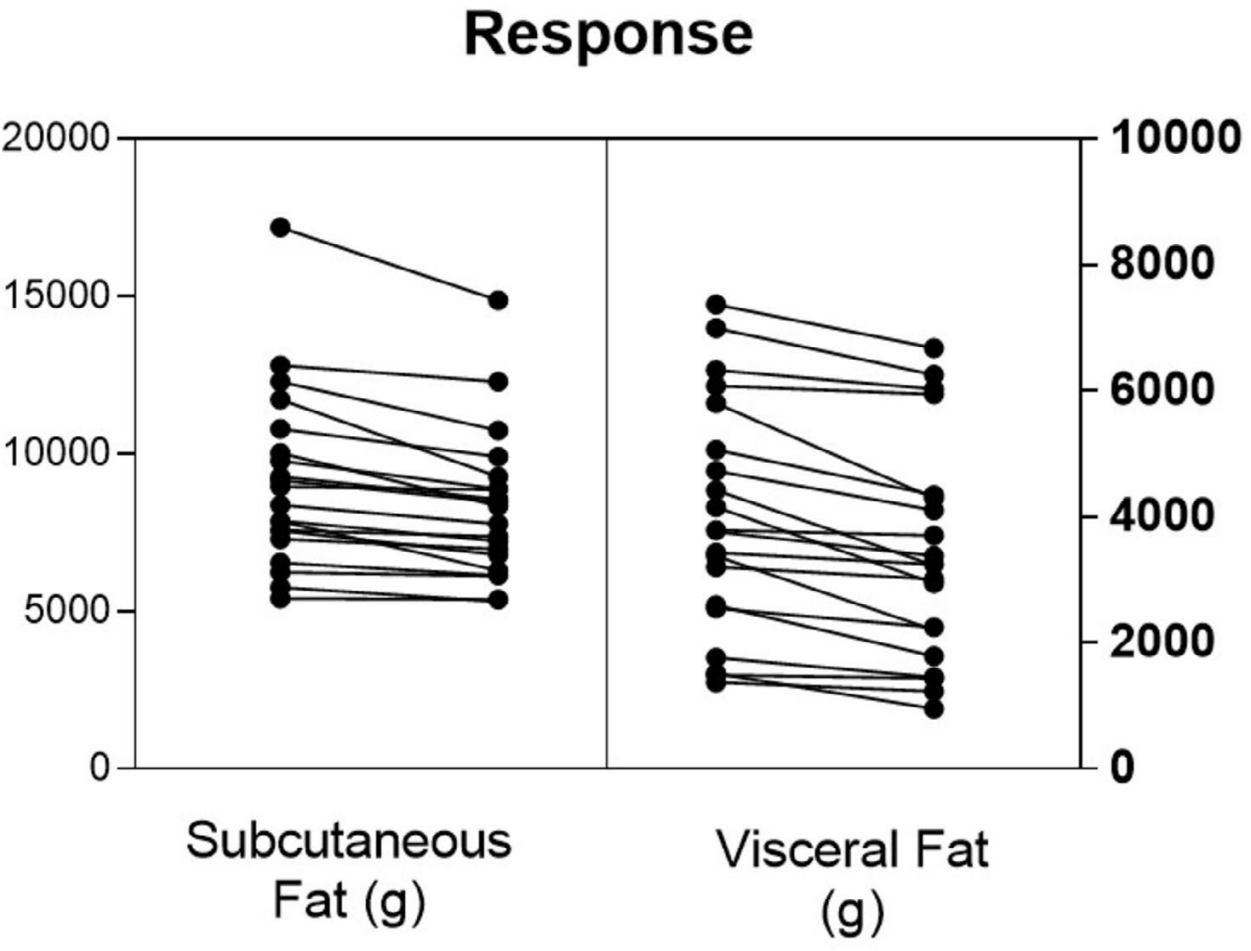
Individual changes in visceral adipose tissue and subcutaneous adipose tissue at baseline and at the end of the radiofrequency period.

The anthropometric data before and after the 10 sessions are shown in Table 1, highlighting significant reductions in waist circumference, waist‐to‐hip ratio, and VAT/SAT volumes. Individual VAT and SAT changes are shown in Figures 2 and 3.

**TABLE 1:** Anthropometry and visceral and subcutaneous fat before and after the procedure.

	Baseline	After treatment	*p*
Height (cm)	166 ± 8.44	166 ± 8.44	0,874
Weight (Kg)	93.9 ± 17.2	94.3 ± 23.6	0.874
BMI (kg/mt^2^)	26.9 ± 4.11	26.7 ± 6.56	0.921
Waist (cm)	109 ± 11.3	103 ± 12.0	0.001
Waist to hip ratio (cm/cm)	1.39 ± 2.07	0.91 ± 0.11	0.048
Vat (cm^3^)	4432 ± 2056	3804 ± 1953	0,001
Vat (g)	3988 ± 1850	3423 ± 1757	0.000
Sat (cm^3^)	10 045 ± 3105	9242 ± 2613	0.001
Sat (g)	9124 ± 2804	8279 ± 2371	0.000
Systolic pressure (mmHg)	139 ± 20.7	124 ± 20.6	0.000
Diastolic pressure (mmHg)	86.1 ± 13.2	85.5 ± 12.0	0.180

Abbreviations: NS, not significative (*p* > 0.05); SAT, subcutaneous abdominal fat; VAT, visceral abdominal fat.

Table 2 shows analytical parameters before and after treatment. Significant decreases were observed in leptin, fasting insulin, and HOMA‐IR (*p* < 0.05).

**TABLE 2:** Analytical data before and after the procedure. Data are shown as mean ± SD or median (IQR).

	Baseline	After treatment	*p*
Glucose (mg/dL)	97 ± 12	93 ± 11	0.063
Cholesterol (mg/dL)	201 ± 52	186.3 ± 52.25	0.268
Triglycerides (mg/dL)	121 ± 35	106.1 ± 48.8	0.166
HDL cholesterol (mg/dL)	46 ± 11	45 ± 12	0.476
LDL cholesterol (mg/dL)	131 ± 41	124 ± 44	0.617
Adiponectin (ng/mL)	5839 ± 1720	5542 ± 1831	0.210
Leptin (ng/mL)	27.5 ± 16	20.2 ± 14.0	0.000
Resistin (ng/mL)	7.3 ± 2.8	7.1 ± 3.2	0.489
TNFα (pg/mL)	6.0 ± 3.1	6.7 ± 3.1	0.239
CRP (mg/L)	4.54 ± 3.13	4.67 ± 3.12	0.857
IL‐6 (pg/mL)	1.90 (1.14–2.87)	1.95 (1.29–2.58)	0,299
Insulin (μIU/mL)	2.31 (1.44–6.49)	1.36 (1.17–2.62)	0.006
HOMA – IR (mg*mU/dL*L)	0.50 (0.32–1.71)	0.33 (0.25–0.63)	0.005

Abbreviations: CRP, C reactive Protein; HDL, high density lipoprotein, HOMA‐IR, Homeostatic Model Assessment of Insulin Resistance; IL‐6, Interleukin 6; LDL, Low density lipoprotein; NS, not significative (*p* ≥ 0.05); SD, Standard deviation; TNFα, Tumor necrosis factor Alpha.

## 4 Discussion

Our study confirms that, in patients with abdominal obesity, the application of 10 sessions with a 1240 W high‐dose treatment reduces not only the SAT but also the VAT, and that this translates into metabolic benefit by reducing serum leptin levels and insulin resistance. This metabolic improvement represents an important novelty in the field. In addition, no deleterious effects or signs of systemic inflammation were observed in the short term.

At 1 MHz, fat tissue activates calcium channels that allow Ca^2+^ to enter, leading to programmed cell death (apoptosis) rather than necrosis [19]. This explains why inflammatory markers remained unchanged in our study and why fat loss occurred, as reflected by the decrease in leptin. Moreover, at this frequency, the heat generated by the electromagnetic field is less dissipated through the skin, resulting in higher temperatures in deeper fat layers. This device, with a larger treatment area and more controlled power, exposes adipocytes to sufficient heat to trigger apoptosis. This factor may largely account for the similarity between our findings and previously reported studies [7], despite the use of different radiofrequency devices and adjunctive exercise. It may also explain the discrepancies observed when comparing our results with other published studies [10, 20, 21, 22, 23] which used lower‐power equipment, lower operating frequencies, or smaller treatment areas.

Although visceral fat loss is important, some subjects had less visceral fat loss. This could be because they have thicker subcutaneous fat with more fibrous tissue, which absorbs more energy and modifies the transmission of electric current. Fibrous septa aligned with the local electric field have a higher absorbed power density than septa oriented perpendicular to the electric field. This can prevent the penetration of sufficient energy into the visceral zone to produce the poration effect in the adipocyte [19].

The reduction of VAT and SAT at the abdominal level in our patients was accompanied by changes in some biochemical parameters. Specifically, there was a significant reduction in insulin levels and a trend towards lower fasting blood glucose values; as a result, insulin resistance measured as HOMA‐IR was significantly reduced (*p* < 0.05). This effect is positive, as rising insulin resistance is associated with the development of high blood pressure, glucose intolerance, and diabetes [20]. In addition, a significant decrease in serum leptin levels was observed, as expected. Serum leptin concentration is directly proportional to the amount of adipose tissue in the body, and when obese people lose fat mass, leptin levels decrease. We did not observe changes in adiponectin (an adipocytokine that is reduced when obesity is associated with insulin resistance, particularly in type 2 diabetes) or in resistin [24].

The decreases in leptin, insulin, HOMA‐IR, and systolic blood pressure give this procedure advantages over surgical procedures for fat removal for which, as we noted, there is no agreement that they improve metabolic indices [23, 24, 25, 26].

Regarding treatment safety, no systemic complications have been reported. However, adverse effects of heat on the skin of varying intensity have been described with radiofrequency treatment, in some cases related to operator error or failure of the device itself [23], but in general, they are well tolerated, with mild and transient adverse effects such as erythema, local discomfort, or transient dysesthesia. Paradoxical adipose hyperplasia has been described exclusively after cryolipolysis for localized fat reduction. To date, there are no reports in the medical literature of paradoxical adipose hyperplasia associated with noninvasive radiofrequency devices [27]. Our data indicate that the reductions in abdominal VAT and SAT achieved with EBD were not accompanied by local inflammatory reactions, as no patients reported inflammatory symptoms at the treatment sites. Likewise, systemic inflammatory markers, IL‐6, TNF‐α, and hs‐CRP, remained unchanged after treatment.

Our findings expand the evidence on non‐invasive energy interventions by showing quantifiable MRI‐detected changes in a very short period, coupled with a novel metabolic improvement. When comparing magnitude and timing with microwave studies [25], ultrasound [23], and studies of natural compounds [4, 5], consistency in acute effects is observed; although persistence and long‐term clinical relevance require controlled trials and prolonged follow‐up.

This study has several strengths, including the use of a relatively homogeneous sample of men and women with abdominal obesity and the reliable assessment of fat changes by MRI.

However, our work has several limitations: this pilot study is a single‐center pre‐post exploratory trial with no control group, a limited sample size, and no psychological assessment. Additional limitations are the short follow‐up period (2 weeks) and the exclusion of common comorbidities that frequently accompany abdominal obesity and insulin resistance, such as hypertension and type 2 diabetes. Therefore, the results should be interpreted as preliminary evidence justifying future randomized trials with prolonged follow‐up.

In conclusion, 1240 W high‐dose multichannel EBD devices appear to be a safe and effective modality to reduce abdominal SAT and VAT, and are accompanied by a clear improvement in the metabolic profile. These findings, together with previously discussed data, provide a new perspective on RF‐based treatment of abdominal obesity and support the need for larger randomized trials to confirm these additional benefits.

## Author Contributions

Jesus Rodríguez‐Lastra, Javier Espíldora‐Hernández and Miguel Ángel Sánchez‐Chaparro conceived the experiments. Sidra Kouser, José Rioja, Rosa María Ordóñez‐Simón, Antonia Blanch, Santiago Mourelo and José Antonio Romero carried out experiments and measurements. Jesus Rodríguez‐Lastra, Javier Espíldora‐Hernández and Miguel Ángel Sánchez‐Chaparro performed all statistical analyses. Miguel Ángel Sánchez‐Chaparro and Javier Espíldora‐Hernández contributed equally to this work. All authors are involved in both writing the paper and the final approval of the submitted and published versions.

## Funding

This study has been financed by CAPENERGY MEDICAL, SL (Barcelona, Spain).

## Ethics Statement

The study was approved by the Ethics Commission in Animal and Human Experimentation (CEEAH) of the Autonomous University of Barcelona (Ref. 5324). All participants provided written informed consent. Patients or members of the public were not involved in the design, conduct, reporting, or dissemination plans of this research.

## Conflicts of Interest

Dr. Jesús Rodriguez Lastra has been Speaker at CAPENERGY MEDICAL, SL. The Lipids and Atherosclerosis Laboratory has been paid by CAPENERGY MEDICAL, SL. under a contracted relationship across the Research Results Transfer Office (OTRI) of the University of Malaga, for having performed the laboratory assays. The remaining authors have no conflicts of interest to declare.

## Data Availability

De‐identified participant data, statistical code, and analytical outputs are available from the corresponding author upon reasonable request.
